# The role of objecthood and animacy in apparent movement processing

**DOI:** 10.1093/scan/nsad014

**Published:** 2023-03-11

**Authors:** Emiel Cracco, Tilia Linthout, Guido Orgs

**Affiliations:** Department of Experimental Clinical and Health Psychology, Ghent University, Ghent 9000, Belgium; Department of Experimental Clinical and Health Psychology, Ghent University, Ghent 9000, Belgium; Department of Psychology, Goldsmiths, University of London, London SE14 6NW, UK; Department of Music, Max Planck Institute for Empirical Aesthetics, Frankfurt 60322, Germany

**Keywords:** apparent movement processing, biological motion perception, objecthood, animacy, frequency tagging

## Abstract

Although the ability to detect the actions of other living beings is key for adaptive social behavior, it is still unclear if biological motion perception is specific to human stimuli. Biological motion perception involves both bottom-up processing of movement kinematics (‘motion pathway’) and top-down reconstruction of movement from changes in the body posture (‘form pathway’). Previous research using point-light displays has shown that processing in the motion pathway depends on the presence of a well-defined, configural shape (objecthood) but not necessarily on whether that shape depicts a living being (animacy). Here, we focused on the form pathway. Specifically, we combined electroencephalography (EEG) frequency tagging with apparent motion to study how objecthood and animacy influence posture processing and the integration of postures into movements. By measuring brain responses to repeating sequences of well-defined or pixelated images (objecthood), depicting human or corkscrew agents (animacy), performing either fluent or non-fluent movements (movement fluency), we found that movement processing was sensitive to objecthood but not animacy. In contrast, posture processing was sensitive to both. Together, these results indicate that reconstructing biological movements from apparent motion sequences requires a well-defined but not necessarily an animate shape. Instead, stimulus animacy appears to be relevant only for posture processing.

## Introduction

Processing other people’s movements is imperative for adaptive social behavior (Pavlova, 2012). Models of biological motion perception posit that there are at least two pathways involved in this process (Giese and Poggio, 2003; Lange and Lappe, 2006), a ‘motion’ and a ‘form’ pathway. Although both these pathways lead to the same biological motion percept, they differ in how this percept is generated. In the motion pathway, it is generated bottom-up by processing the kinematics of the observed movement (e.g. Grossman and Blake, 2001; Troje, 2013). In the form pathway, it is instead generated top-down by reconstructing the movement based on changes in body posture (e.g. Shiffrar and Freyd, 1990; Orgs *et al.*, 2011).

To dissociate between both pathways, specific stimuli have been developed. Processing in the motion pathway is typically studied using point-light figures, which present human or animal movements as a constellation of dots or lines (e.g. Johansson, 1973; Blake and Shiffrar, 2007). By stripping away most form information, these stimuli primarily convey motion information and therefore target the processing of biological movement kinematics (Giese and Poggio, 2003; Troje, 2013). Processing in the form pathway has instead been studied using apparent motion stimuli. These stimuli present movements as a sequence of static images and thereby strip away motion information (e.g. Shiffrar and Freyd, 1990; Orgs *et al.*, 2011). Despite the absence of retinal motion in such sequences, studies have shown that they induce a vivid motion percept as long as the interval between the images is consistent with the duration of the implied movement (Shiffrar and Freyd, 1990; Grosjean *et al.*, 2007).

Motion- and form-based processing of biological movements have shown to rely on distinct neural mechanisms. Whereas motion information is processed mainly in motion-specific areas such as the middle temporal cortex (Vaina *et al.*, 2001; Mather *et al.*, 2016), form information is primarily processed in body-specific areas such as the extrastriate body area (Vaina *et al.*, 2001; Orgs *et al.*, 2016). Eventually, however, both types of information converge in higher-order areas like the superior temporal sulcus (Vaina *et al.*, 2001; Pitcher and Ungerleider, 2021).

What is less clear is whether processing along the motion and form pathways is specific to biological shapes. Jastorff *et al.* (2006, 2009) used point-light stimuli to investigate this question in the motion pathway. More specifically, they measured the processing of biological (human) and non-biological (artificial) point-light stimuli before and after training participants to discriminate among different stimulus exemplars. The results revealed that, after training, non-biological stimuli were processed in the same way as biological stimuli, as long as they had a clear underlying shape (as opposed to being scrambled). In other words, non-biological stimuli were processed similarly to biological stimuli as long as they qualified as well-defined, configural objects. This suggests that processing in the motion pathway does not rely on animacy (i.e. whether the shape depicts a living being) but on objecthood more generally (i.e. whether the shape represents a well-defined, recognizable object), and hence that animacy is a result, not a prerequisite, of motion processing.

Research on the form pathway suggests that it is likewise affected by objecthood manipulations, such as pixelation (Orgs *et al.*, 2011, 2016) or inversion (Cracco *et al.*, 2022a). However, whether processing in this pathway is affected by animacy manipulations is not yet known. On the one hand, a biological shape may be necessary because the form pathway involves brain areas known to display biological specificity (Downing, 2001; Orgs *et al.*, 2016). On the other hand, recent work suggests that even in those brain areas, animacy is not represented categorically but on a continuum from more to less alive (Sha *et al.*, 2015; Thorat *et al.*, 2019). Here, we used a recently developed frequency tagging task to directly test if apparent movement perception is specific to biological shapes or generalizes to non-biological shapes as well (Cracco *et al.*, 2022a). Frequency tagging is a technique to isolate the processing of a particular stimulus by presenting this stimulus at a fixed frequency and analyzing only the brain responses that occur at the same frequency (for a review, see Norcia *et al.*, 2015). For example, in a study on face perception, Rossion *et al.* (2012) found that presenting a face every 250 ms (i.e. at 4 Hz) elicited a 4 Hz brain response that was sensitive to two manipulations known to affect face processing: inversion and contrast-reversal.

**Fig. 1.**
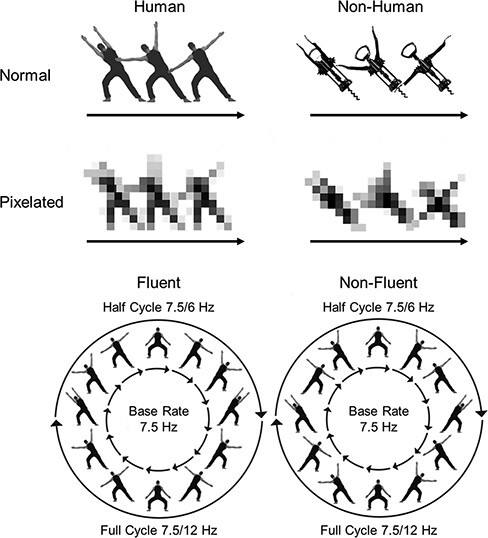
Stimuli and paradigms. The top shows the four types of stimuli used in the study. The bottom shows the structure of the fluent and non-fluent sequences. The latter figure is adapted from Figure 2 of Cracco *et al.* (2022a), where it was published under a CC BY license.

Because frequency tagging confines the brain response to a specific frequency, it provides both an objective and sensitive measure of stimulus processing (Norcia *et al.*, 2015). As a result, it has become a popular tool in visual neuroscience, with successful applications in various domains, including tool processing (De Keyser *et al.*, 2018), visual perspective taking (Beck *et al.*, 2018) and interaction perception (Oomen *et al.*, 2022), among others. More recently, it has also been used to study biological movement processing, both in the motion pathway (Cracco *et al.*, 2022b) and in the form pathway (Cracco *et al.*, 2022a). In the form pathway task, a cyclical 12-image apparent motion sequence is shown repeatedly using a fixed image presentation rate (Figure 1). Importantly, the sequence is symmetric, with the second half of the images mirroring the first half. As a result, three brain responses are elicited: a brain response coupled to the frequency of image presentation (base rate), a brain response coupled to the symmetry point in the image sequence (half-cycle rate) and a brain response coupled to the completion of the entire sequence, that is image repetition (full-cycle rate).

To measure apparent movement processing, two types of sequences are compared: fluent sequences and non-fluent sequences. In fluent sequences, the images are ordered to produce the percept of a human agent making fluent movements. In non-fluent sequences, they are instead ordered to disrupt this percept. Importantly, in the fluent condition, movements coincide with the half cycle symmetry point. Cracco *et al.* (2022a) found that this made the symmetry point more salient, leading to stronger half-cycle responses. In contrast, full-cycle and base rate responses both showed the opposite pattern, with stronger responses for non-fluent than for fluent sequences. In line with evidence that scrambling the image order of an apparent motion sequence causes image processing to take over (e.g. Downing *et al.*, 2006), this indicates that non-fluent sequences were processed as ‘image sequences’ at base rate (presentation of images) and full-cycle rate (completion of an image sequence), whereas fluent sequences were processed as ‘movement sequences’ at half-cycle rate. Stated differently, it indicates that stronger half-cycle responses for fluent *vs* non-fluent sequences can be taken as an index of movement processing, whereas stronger full-cycle and base rate responses for non-fluent *vs* fluent sequences can be taken as an index of image processing.

Here, we use the frequency tagging task developed by Cracco *et al.* (2022a) to investigate what drives biological motion processing along the form pathway: objecthood, animacy or both? To this end, we combined a fluency manipulation (fluent *vs* non-fluent movements) with objecthood (normal *vs* pixelated images) and animacy (human *vs* corkscrew) manipulations (Figure 1). If processing in the form pathway is driven not just by objecthood (e.g. Orgs *et al.*, 2011, 2016) but also by animacy, we should find an interaction between pixelation and animacy on half-cycle amplitudes, such that half-cycle responses are strongest for sequences showing non-pixelated human images. Moreover, if these effects are specific to movement processing, we should further find that they are stronger for fluent than for non-fluent sequences, given that only fluent sequences produce a movement percept (Cracco *et al.*, 2022a). Although we also measured full-cycle and base rate responses, we had no clear a priori predictions for these responses.

## Methods

### Participants

As this is the first study to investigate the influence of objecthood and animacy on apparent movement processing, we did not have strong expectations about the anticipated effect size. However, previous research using the same task found medium-to-large effect sizes (i.e. *d*_z_ > 0.60) on half-cycle responses for manipulations of fluency, also included here, and inversion, a manipulation of stimulus shape. Therefore, we conservatively assumed a medium-sized effect size (*d*_z_ = 0.50). An a priori power analysis revealed that we needed at least 33 participants to detect such effect sizes with 80% power. Unfortunately, after testing the pre-registered sample of *N* = 33, we discovered an undetected technical issue that resulted in bad data quality for 11 participants (i.e. >10% of the electrodes requiring interpolation). Although we had not pre-registered to compensate for excluded participants, the current study was conducted together with another study where we had pre-registered to add three more participants if the sample size dropped below 30, until the sample size after exclusions was at least 30 (Cracco *et al.*, 2022b; pre-registration: https://aspredicted.org/1P9_PNW). In that study, nine participants had to be excluded, and hence six participants were compensated. Given the large number of exclusions also in the current study, we decided to again combine both tasks for these six participants. This resulted in an eventual sample size of 28 (9 males and 19 females; *M*_age_ = 23.14 years, range_age_ = 18–33 years). All task procedures were conducted in accordance with the ethical protocol of the Faculty of Psychological and Educational Sciences at Ghent University.

### Task, stimuli and procedure

**Fig. 2.**
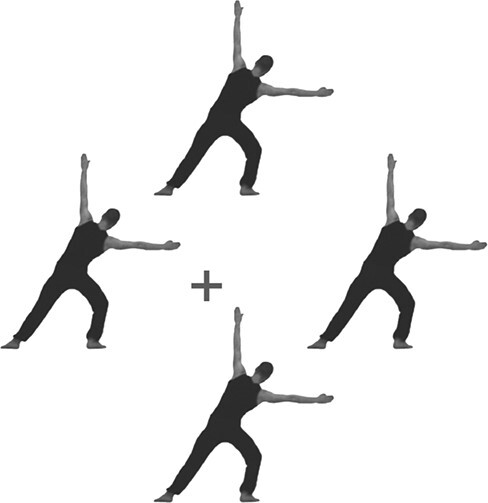
An example frame. Participants watched short videos showing four agents arranged symmetrically around a fixation cross, synchronously performing identical movements. Participants had to focus on the fixation cross and press the space bar each time it turned red. See https://doi.org/10.17605/OSF.IO/JNTQZ for example videos.

Participants were seated in a Faraday cage, approximately 80–100 cm from a 24-inch computer monitor with a 60 Hz refresh rate. The experiment was programmed in PsychoPy (Peirce *et al.,* 2019) and was based on Experiments 2–3 of Cracco *et al.* (2022a). That is, participants watched videos of four identical agents arranged symmetrically around a fixation cross, synchronously performing the same movements (Figures 1–2). Note that four agents were shown instead of one to avoid overlap between the fixation cross and the moving stimuli. To control eye gaze and attention, participants were asked to focus on this fixation cross and to press the space bar every time it briefly (∼400 ms) turned red. Movements were presented as a repeating sequence of 12 images, rendered on the screen at a rate of 7.5 Hz. All sequences had the same symmetrical structure, with the second half of the sequence mirroring the first half. Previous research has shown that this elicits three brain responses: a brain response at 7.5 Hz (base rate), coupled to image presentation, a brain response at 1.25 Hz (half-cycle rate), coupled to the symmetry point in the sequence, and a brain response at 0.625 Hz (full-cycle rate), coupled to the repetition of the full image sequence (Cracco *et al.*, 2022a).

Movement fluency was manipulated by changing the order of image presentation. In the fluent condition, images were ordered to depict the agents performing a rhythmical dance movement that involved moving their arms from left to right and back from right to left. In the non-fluent condition, images were rearranged to break this movement and to maximize visual displacement between body postures (Figure 1). Although fluent and non-fluent sequences had the same symmetrical structure, this structure was more salient in the fluent condition, because in that condition the half-cycle symmetry point coincided with movement completion. In the fluent condition, the half-cycle point thus signaled a change in the movement direction (i.e. from left to right or from right to left) and half-cycle amplitudes captured movement processing. In contrast, base rate amplitudes and full-cycle amplitudes captured image processing (Cracco *et al.*, 2022a).

In addition to movement fluency (fluent or non-fluent), we also manipulated stimulus animacy (human or corkscrew) and objecthood (well-defined or pixelated shape), resulting in eight experimental conditions, presented in random order as separate videos, with three repetitions per condition. Each video lasted for 60 sequence repetitions (i.e. 96 s), including a 3.2-s fade-in and fade-out to minimize eye blinking. Pixelated versions of the human and corkscrew images were created by degrading the pixel size with a 50 × 50 pixel size. All stimuli were matched for size as well as for contrast and luminance using the SHINE toolbox in Matlab 2020a. Nevertheless, it is not possible to fully match different stimuli for their apparent movement paths because the path is fully determined by the shape and position of the stimulus. To compare the saliency of visual change between human, corkscrew and pixelated image sequences, we conducted a visual change analysis comparing the average pixel change between adjacent images (e.g. Orlandi *et al.*, 2020; Table 1). This revealed that human and corkscrew stimuli were relatively matched for the saliency of image transitions, but that image transitions were more salient for pixelated *vs* normal sequences and to a lesser extent for non-fluent *vs* fluent sequences. The visual change analysis thus indicated that stronger brain responses for pixelated and non-fluent sequences could be driven by increased stimulus flicker.

**Table 1.** Average pixel change between adjacent images

	Normal		Pixelated	
	Human	Corkscrew	Human	Corkscrew
Fluent	1.35	1.39	6.08	5.61
Non-fluent	1.87	1.62	8.46	6.67

### EEG recording and pre-processing

The electroencephalography (EEG) signal was recorded from 64 Ag/AgCl (active) electrodes using an actiCHamp amplifier and BrainVision Recorder software (version 1.21.0402; Brain Products, Gilching, Germany) at a sampling rate of 1000 Hz. Electrodes were positioned according to the 10% system, except for two electrodes (TP9 and TP10), which were placed at OI1h and OI2h according to the 5% system to have better coverage of posterior scalp sites. Fpz was used as the ground electrode and Fz as the online reference. Horizontal eye movements were recorded with two electrodes embedded in the EEG cap (FT9 and FT10) and vertical eye movements with two additional bipolar Ag/AgCl sintered ring electrodes placed above and below the left eye.

Letswave 6 (www.letswave.org) was used for offline processing of the data. First, a fourth-order Butterworth bandpass filter with cut-off values 0.1 Hz and 100 Hz was applied. Next, the data were segmented according to the experimental conditions, and ocular artifacts were removed using independent component analysis (ICA; RUNICA algorithm, square mixing matrix). After ICA, faulty or excessively noisy electrodes (2.8% on average, never >10%) were interpolated from the three closest neighbors, and the data were re-referenced to the average signal across electrodes. Finally, the fade-in and fade-out for each trial were cropped from the re-referenced epochs and averaged per condition before transforming them to normalized (divided by N/2) amplitudes (μV) in the frequency domain using a fast Fourier transform.

### Data analysis

Brain responses at base rate (7.5 Hz), half cycle (1.25 Hz) and full cycle (0.625 Hz) were calculated as in Cracco *et al.* (2022a). Importantly, frequency tagging elicits a brain response not only at the tagged frequency (F) but also at multiples of this frequency (2 F, 3 F, …). As the brain response is distributed across these harmonics (Retter *et al.*, 2021), we considered the first 10 harmonics per response. More specifically, we first baseline-corrected amplitudes at each frequency bin by subtracting the signal from the 10 neighboring frequency bins on each side (excluding directly adjacent bins) and then summed the first 10 relevant harmonics per response, excluding those harmonics that overlapped with the harmonics of a higher frequency response (as recommended by Retter *et al.*, 2021). The base rate harmonics included 7.5, 15, 22.5, 30, 37.5, 45, 52.5, 60, 67.5 and 75 Hz. The half-cycle harmonics included 1.25, 2.50, 3.75, 5.00, 6.25, 8.75, 10, 11.25, 12.50 and 13.75 Hz. Finally, the full-cycle harmonics included 0.625, 1.875, 3.125, 4.375, 5.625, 6.875, 8.125, 9.375, 10.625 and 11.875 Hz.

**Fig. 3.**
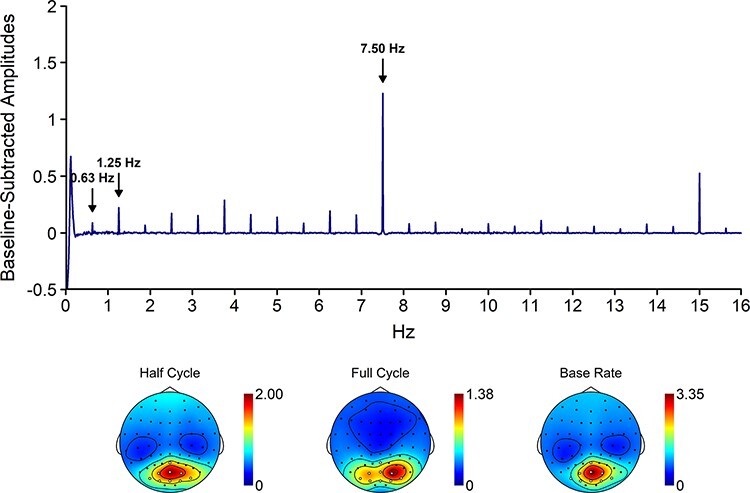
The collapsed spectrum plot and topographies of the baseline-subtracted amplitudes. The spectrum plot shows the signal across the electrodes included into the analysis. The electrodes included in the analysis are marked in white on the topographies. The topographies are scaled from 0 to the maximum value across the entire scalp for the respective response.

As in Cracco *et al.* (2022a), we initially defined four electrode clusters to include in the analysis: a left posterior cluster (PO3, PO7, O1), a middle posterior cluster (Oz, OI1h, OI2h), a right posterior cluster (PO4, PO8, O2) and a middle frontocentral cluster (FCz, FC1, FC2). However, we decided to deviate from this pre-registered approach because a collapsed localizer revealed that (i) there was no clear frontocentral activation in the current study and (ii) the middle posterior cluster was not well captured by the pre-registered electrodes (Figure 3).^1^ Accordingly, we did not include a frontocentral cluster in the analyses and used Oz, POz and Pz to capture the middle posterior cluster rather than Oz, OI1h and OI2h. The brain response in each cluster was quantified by averaging the response of the included electrodes.

The data were analyzed with a cluster (left, middle or right posterior) × fluency (non-fluent or fluent) × pixelation (pixelated or normal) × animacy (corkscrew or human) Bayesian repeated-measures analysis of variance (ANOVA) separately for half-cycle, full-cycle and base rate amplitudes (Rouder *et al.*, 2012). Note that we had initially planned to do a frequentist ANOVA but decided to instead conduct a more conservative Bayesian ANOVA to deal with the heightened false-positive rate due to the large number of statistical tests. In addition, to simplify the results and because we were not interested in cluster effects, we added the main effect of the cluster to the null model and did not fit interactions with the cluster. Note, however, that the results did not change if we did fit these interactions, as shown in Supplementary Material. The Bayesian ANOVA was performed in JASP (JASP Team, 2022, v0.16.3) with default priors and using a model-averaging approach that compared all models with the effect of interest to matched models without the effect of interest, excluding higher-order interactions (Wagenmakers *et al.*, 2018). This yields a Bayes factor (BF) for each effect, which represents the likelihood of the data under models containing *vs* not containing the corresponding effect. A BF >3 is typically considered reliable evidence for the presence of an effect, whereas a BF <3 is typically considered reliable evidence against the presence of an effect (Jeffreys, 1961). If the Bayesian ANOVA revealed evidence for an effect with more than two levels, we followed up on this effect using Bayesian paired *t*-tests with default JASP priors. In addition to the BF, we also reported Cohen’s *d*_z_ to further quantify the size of each effect (Lakens, 2013).

## Results

### Half-cycle rate

**Fig. 4.**
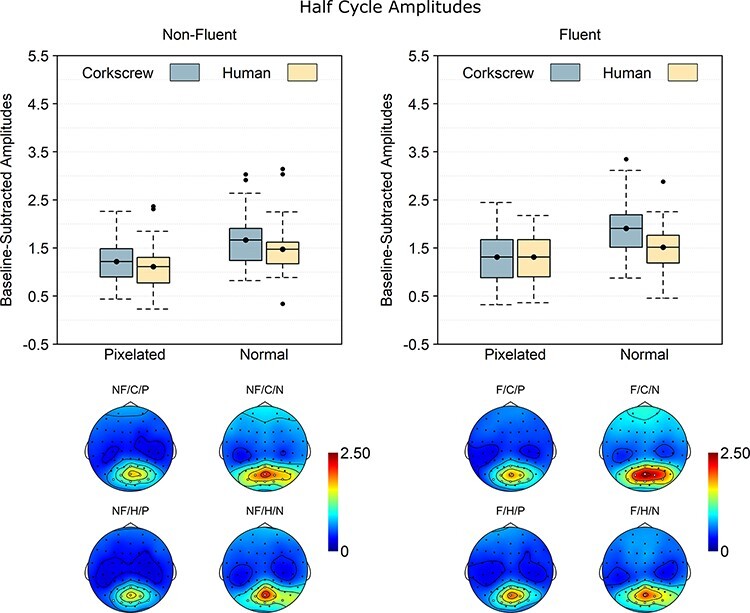
Baseline-subtracted amplitudes at half-cycle rate (1.25 Hz) and its harmonics. Boxplots show the mean instead of the median to better match the statistical analysis. Note that 0 is the baseline and that values below 0 necessarily reflect noise. The dots going beyond the whiskers are data points exceeding the first or third quartile by more than 1.5 times the interquartile range. The topographies are scaled from 0 to the maximum value across the scalp and across the different conditions. NF: non-fluent; F: fluent; C: corkscrew; H: human; P: pixelated; N: normal.

The brain response at half-cycle rate is coupled to the symmetry point in the sequences. In fluent sequences, this corresponds to the completion of a body movement. Confirming that half-cycle responses captured movement processing, the analysis of the half-cycle response (Figure 4) revealed a main effect of fluency, BF_10_ = 10.54, d_z_ = 0.60, with stronger responses for fluent than for non-fluent sequences. In addition, there was a main effect of pixelation, BF_10_ = 1.45 × 10^7^, *d*_z_ = 1.74, with stronger responses for normal than for pixelated stimuli, and a main effect of animacy, BF_10_ = 109.52, *d*_z_ = 0.84, with stronger responses for corkscrews than for humans. The main effect of animacy was further qualified by a pixelation × animacy interaction, BF_10_ = 4.66, *d*_z_ = 0.51, indicating that the animacy effect was reliable for normal, BF_10_ = 1.97 × 10^3^, *d*_z_ = 1.02, but not for pixelated images, BF_10_ = 0.28, *d*_z_ = 0.16. No evidence was found for any of the other interaction effects, 0.23 ≤ BF_10_ ≤ 1.32.

### Full-cycle rate

**Fig. 5.**
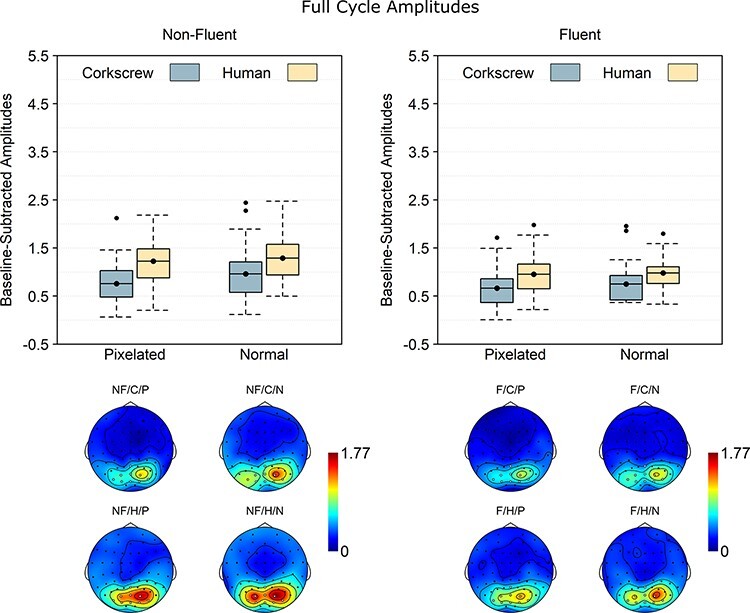
Baseline-subtracted amplitudes at full-cycle rate (0.625 Hz) and its harmonics. Boxplots show the mean instead of the median to better match the statistical analysis. Note that 0 is the baseline and that values below 0 necessarily reflect noise. The dots going beyond the whiskers are data points exceeding the first or third quartile by more than 1.5 times the interquartile range. The topographies are scaled from 0 to the maximum value across the scalp and across the different conditions. NF: non-fluent; F: fluent; C: corkscrew; H: human; P: pixelated; N: normal.

The brain response at full-cycle rate is coupled to the completion of the full image sequence. In line with the half-cycle analysis, the full-cycle analysis (Figure 5) revealed main effects of fluency, BF_10_ = 1.38 × 10^5^, *d*_z_ = 1.38, pixelation, BF_10_ = 10.97, *d*_z_ = 0.64, and animacy, BF_10_ = 6.59 × 10^3^, *d*_z_ = 1.11. However, whereas the pixelation effect mirrored the half-cycle results (normal > pixelated), the fluency (non-fluent > fluent) and animacy effects (human > corkscrew) were opposite. No evidence was found for any of the other effects, 0.44 ≤ BF_10_ ≤ 1.11.

### Base rate

**Fig. 6.**
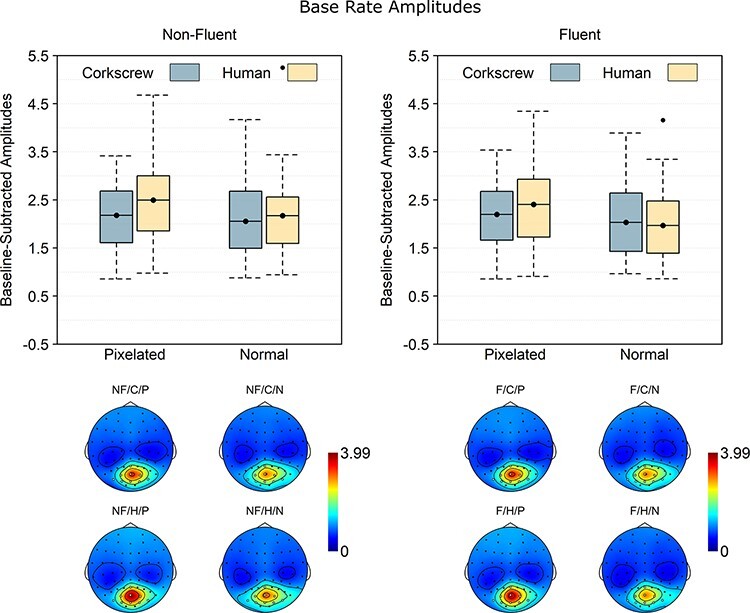
Baseline-subtracted amplitudes at base rate (7.5 Hz) and its harmonics. Boxplots show the mean instead of the median to better match the statistical analysis. Note that 0 is the baseline and that values below 0 necessarily reflect noise. The dots going beyond the whiskers are data points exceeding the first or third quartile by more than 1.5 times the interquartile range. The topographies are scaled from 0 to the maximum value across the scalp and across the different conditions. NF: non-fluent; F: fluent; C: corkscrew; H: human; P: pixelated; N: normal.

The brain response at base rate is coupled to the presentation of the individual images. The base rate analysis (Figure 6) revealed reliable main effects of fluency, BF_10_ = 3.28, *d*_z_ = 0.52, and pixelation, BF_10_ = 69.20, *d*_z_ = 0.76, but not animacy, BF_10_ = 2.90, *d*_z_ = 0.45. Interestingly, while the fluency effect (non-fluent > fluent) mirrored the full-cycle response, the pixelation effect was opposite (pixelated > normal). In addition to these main effects, there was also a pixelation × animacy interaction, BF_10_ = 6.11, *d*_z_ = 0.54, indicating that that the pixelation effect was reliable for humans, BF_10_ = 1.06 × 10^3^, *d*_z_ = 0.97, but not for corkscrew sequences, BF_10_ = 0.83, *d*_z_ = 0.34. No evidence was found for any of the other effects, 0.41 ≤ BF_10_ ≤ 1.68.

## Discussion

This study investigated the influence of objecthood and animacy on movement processing in the form pathway. To this end, we showed normal and pixelated image sequences of human and corkscrew stimuli producing fluent and non-fluent apparent movements. These movements occurred at half the rate of sequence presentation, eliciting corresponding brain responses that can be used as a measure of movement processing (Cracco *et al.*, 2022a). Supporting the hypothesis that objecthood would influence movement processing, we found weaker half-cycle responses for pixelated than for non-pixelated stimuli. In contrast, we found no evidence for biological specificity, with stronger as opposed to weaker responses for corkscrew than for the human stimuli. Finally, although we found overall stronger half-cycle responses for fluent than for non-fluent sequences (Cracco *et al.*, 2022a), neither the objecthood nor the animacy effect depended on movement fluency.

The absence of interactions with movement fluency indicates that neither the effect of objecthood nor the effect of animacy was specific to movement processing. Indeed, scrambling the order of images in an apparent movement sequence is well known to disrupt movement processing (Orgs *et al.*, 2016; Cracco *et al.*, 2022a), as it makes it more difficult to integrate images over time into a coherent movement percept (Lange and Lappe, 2006, 2007). Importantly, however, binding postures into meaningful chunks is not necessarily unique to movement processing and postures could also be structured according to different criteria. For instance, sequences were symmetrical around the half-cycle point in our task. As symmetry is well known to be a principle of perceptual organization (Wagemans *et al.*, 2012), this could have also stimulated posture integration. Applied to objecthood, this would mean that binding stimuli into chunks is easier when they have a configural, well-defined shape (main effect of pixelation) and that this effect is relevant for movement processing (main effect of movement fluency) but not specific to movement processing (no interaction).

Interestingly, this interpretation is at odds with a previous functional magnetic resonance imaging study that did find a movement-specific effect of stimulus pixelation (Orgs *et al.*, 2016). However, movement specificity was limited to motor areas. Brain activity in the current study, on the other hand, was restricted to posterior brain areas. It is, therefore, possible that movement-specific effects of objecthood exist only in the motor cortex. If so, this would suggest that they are driven by motor simulation, the process of mentally simulating other people’s actions in one’s own motor system (Jeannerod, 2001). Although frontal brain activity indicative of motor simulation was observed in three previous experiments using the same paradigm (Cracco *et al.*, 2022a), the same frontal activation cluster was not found here. While speculative, this is potentially the result of mixing sequences with human and non-human agents. Indeed, motor simulation is known to be reduced or absent for non-human agents (Press, 2011; Cracco *et al.*, 2018) and mixing human with non-human sequences may therefore have led to a more perceptual processing style as opposed to previous studies (Orgs *et al.*, 2016; Cracco *et al.*, 2022a).

In contrast to objecthood, stimulus animacy had the opposite effect as predicted, namely stronger responses for the corkscrew than for the human agent. A likely explanation for this finding is that the movements made by the corkscrew stimulus were more salient. This could have had several reasons. First, it is possible that the corkscrew’s movements were more salient because it is unusual to see an inanimate object move independently. Second, consistent with evidence that apparent movement perception depends on the rate at which the images are presented (Shiffrar and Freyd, 1990; Grosjean *et al.*, 2007), the corkscrew’s movements may have been more salient because the presentation rate was more suited for the corkscrew than for the human stimulus. Nevertheless, regardless of the explanation, it is clear that, at the very least, animacy is not the primary force driving movement processing in the form pathway.

In addition to brain responses at half-cycle rate, we also measured two other responses: brain responses at full-cycle rate and brain responses at base rate. In a previous study, we interpreted full-cycle responses as reflecting the processing of body posture sequences and found that they were stronger when the individual postures became more salient, such as in non-fluent sequences (Cracco *et al.*, 2022a). The current results not only replicate this finding but further bolster it by showing that full-cycle responses were also stronger for human than for pixelated and non-human postures. These results are consistent with the previous evidence of dedicated brain areas for processing human body images, such as the fusiform (Schwarzlose *et al.*, 2005) and extrastriate body area (Downing *et al.*, 2006), and suggest that the full-cycle response, unlike the half-cycle response, is more sensitive to human than to non-human stimuli.

The base rate response, because it is coupled to image presentation, was previously interpreted to capture (body) image processing regardless of posture (Cracco *et al.*, 2022a). However, for the same reason, it is also likely to capture changes in contrast from image to image. Supporting the latter view, the base rate response tended to be stronger for sequences where visual change was larger (Table 1). Indeed, both the base rate and visual change analyses found the largest differences between pixelated and normal sequences, then between non-fluent and fluent sequences, and finally between human and corkscrew sequences. It therefore seems likely that base rate responses primarily capture low-level visual processing. This same reasoning also implies that low-level visual processing is not the primary driver of the half- and full-cycle responses since they were both reduced for pixelated stimuli.

In sum, the current study shows that processing in the form pathway involves body specificity when processing individual shapes but not when binding these shapes into a movement percept. Instead, movement perception in the form pathway, like in the motion pathway (Jastorff *et al.*, 2006, 2009), requires a well-defined but not an animate shape. This suggests that biological motion perception relies on domain-general mechanisms that are used not only to process biological movements but also to process other kinds of structured visual sequences.

## Supplementary Material

nsad014_Supp

## Data Availability

This study was pre-registered as ‘Brain representations of human and non-human apparent motion’, available at https://aspredicted.org/8RH_NXF. Deviations from the pre-registration are mentioned and justified in the Methods section. Videos of the stimuli are available at the Open Science Framework, together with the pre-processed data and the analysis script (https://doi.org/10.17605/OSF.IO/JNTQZ).

## Footnotes

1 Note that this is likely because this study uses a different electrode setup that does not include all electrodes included in the middle posterior cluster of the original study of Cracco *et al.* (2022a).
