# Supplementary material for: The role of objecthood and animacy in apparent movement processing
Source: Soc Cogn Affect Neurosci. 2023 Mar 11;18(1):nsad014. doi: 10.1093/scan/nsad014 (PMC10032357; doi:10.1093/scan/nsad014)
Supplement: nsad014_Supp [file nsad014_supp.zip › scan-22-170-File008.docx]

**Supplementary Material**

**Half Cycle Results With Cluster**

**Table S1.** Half Cycle Results Table

| **Effect** | **BF_10_** | **Pattern** |
| --- | --- | --- |
| Cluster | 67.86 | M > L  R > L  M ? R |
| Fluency | 11.85 | F > NF |
| Agent | 117.79 | C > H |
| Pixelation | 1.34x10^7^ | N > P |
| Cluster x Fluency | 0.07 | NA |
| Cluster x Agent | 3.03 | L: C > H  M: C ? H  R: C > H |
| Cluster x Pixelation | 0.17 | NA |
| Fluency x Agent | 0.36 | NA |
| Fluency x Pixelation | 0.30 | NA |
| Agent x Pixelation | 5.47 | P: C = H  N: C > H |
| Cluster x Fluency x Agent | 0.20 | NA |
| Cluster x Fluency x Pixelation | 0.19 | NA |
| Cluster x Agent x Pixelation | 2.30 | NA |
| Fluency x Agent x Pixelation | 0.57 | NA |
| Cluster x Fluency x Agent x Pixelation | 8.19x10^-15^ | NA |

*Note.* BF_10_ ≤ 0.33 is denoted with “=”, 0.33 < BF_10_ < 3 with “?”, and BF_10_ ≥ 3 with “>”. L: left, M: middle, R: right, NF: non-fluent, F: fluent, C: corkscrew, H: human, P: pixelated, N: normal.

**Table S2.** Means and Standard Deviations of the Half Cycle Response

| **Left** | Non-Fluent | | Fluent | |
| --- | --- | --- | --- | --- |
|  | Pixelated | Normal | Pixelated | Normal |
| Corkscrew | 1.01 ± 0.51 | 1.64 ± 0.74 | 1.12 ± 0.65 | 1.80 ± 0.81 |
| Human | 0.98 ± 0.69 | 1.25 ± 0.71 | 1.13 ± 0.62 | 1.35 ± 0.59 |
| **Middle** | Non-Fluent | | Fluent | |
|  | Pixelated | Normal | Pixelated | Normal |
| Corkscrew | 1.37 ± 0.67 | 1.71 ± 0.93 | 1.47 ± 0.83 | 1.97 ± 1.14 |
| Human | 1.29 ± 0.81 | 1.73 ± 1.04 | 1.54 ± 0.92 | 1.67 ± 0.97 |
| **Right** | Non-Fluent | | Fluent | |
|  | Pixelated | Normal | Pixelated | Normal |
| Corkscrew | 1.27 ± 0.58 | 1.64 ± 0.76 | 1.34 ± 0.73 | 1.95 ± 0.92 |
| Human | 1.07 ± 0.72 | 1.44 ± 0.76 | 1.26 ± 0.80 | 1.52 ± 0.72 |

**Full Cycle Results With Cluster**

**Table S3.** Full Cycle Results Table

| **Effect** | **BF_10_** | **Pattern** |
| --- | --- | --- |
| Cluster | 194.80 | R > M  R > L  L ? M |
| Fluency | 1.32x10^5^ | NF > F |
| Agent | 6.60x10^3^ | H > C |
| Pixelation | 10.82 | N > P |
| Cluster x Fluency | 0.10 | NA |
| Cluster x Agent | 3.57x10^-7^ | NA |
| Cluster x Pixelation | 3.60 | L: N > P  M: N = P  R: N ? P |
| Fluency x Agent | 1.14 | NA |
| Fluency x Pixelation | 0.75 | NA |
| Agent x Pixelation | 0.88 | NA |
| Cluster x Fluency x Agent | 9.16x10^-4^ | NA |
| Cluster x Fluency x Pixelation | 0.11 | NA |
| Cluster x Agent x Pixelation | 6.47 | Unclear |
| Fluency x Agent x Pixelation | 0.19 | NA |
| Cluster x Fluency x Agent x Pixelation | 2.00x10^-3^ | NA |

*Note.* BF_10_ ≤ 0.33 is denoted with “=”, 0.33 < BF_10_ < 3 with “?”, and BF_10_ ≥ 3 with “>”. L: left, M: middle, R: right, NF: non-fluent, F: fluent, C: corkscrew, H: human, P: pixelated, N: normal. The pattern underlying the cluster x agent x pixelation interaction suggested the presence of an agent x pixelation interaction for the middle cluster but not for the other two clusters. However, this pattern could not be supported by post-hoc Bayesian paired *t*-tests.

**Table S4.** Means and Standard Deviations of the Full Cycle Response

| **Left** | Non-Fluent | | Fluent | |
| --- | --- | --- | --- | --- |
|  | Pixelated | Normal | Pixelated | Normal |
| Corkscrew | 0.71 ± 0.67 | 0.93 ± 0.78 | 0.63 ± 0.61 | 0.77 ± 0.69 |
| Human | 1.14 ± 0.67 | 1.36 ± 0.77 | 0.90 ± 0.55 | 1.01 ± 0.60 |
| **Middle** | Non-Fluent | | Fluent | |
|  | Pixelated | Normal | Pixelated | Normal |
| Corkscrew | 0.65 ± 0.56 | 0.79 ± 0.67 | 0.57 ± 0.45 | 0.57 ± 0.44 |
| Human | 1.12 ± 0.70 | 1.06 ± 0.81 | 0.86 ± 0.64 | 0.75 ± 0.58 |
| **Right** | Non-Fluent | | Fluent | |
|  | Pixelated | Normal | Pixelated | Normal |
| Corkscrew | 0.91 ± 0.77 | 1.17 ± 0.94 | 0.79 ± 0.67 | 0.91 ± 0.77 |
| Human | 1.43 ± 0.72 | 1.45 ± 0.85 | 1.09 ± 0.65 | 1.17 ± 0.62 |

**Base Rate Results With Cluster**

**Table S5.** Base Rate Results Table

| **Effect** | **BF_10_** | **Pattern** |
| --- | --- | --- |
| Cluster | 3.45x10^4^ | M > L  M > R  R > L |
| Fluency | 4.21 | NF > F |
| Agent | 3.10 | H > P |
| Pixelation | 60.29 | P > N |
| Cluster x Fluency | 0.20 | NA |
| Cluster x Agent | 1.25 | NA |
| Cluster x Pixelation | 29.99 | L: P ? N  M: P > N  R: P > N |
| Fluency x Agent | 1.45 | NA |
| Fluency x Pixelation | 1.53 | NA |
| Agent x Pixelation | 6.72 | C: P ? N  H: P > N |
| Cluster x Fluency x Agent | 0.18 | NA |
| Cluster x Fluency x Pixelation | 0.14 | NA |
| Cluster x Agent x Pixelation | 3.29 | L: Agent x Pixelation ?  M: Agent x Pixelation +  R: Agent x Pixelation ? |
| Fluency x Agent x Pixelation | 0.40 | NA |
| Cluster x Fluency x Agent x Pixelation | 13.78 | Too complex |

*Note.* BF_10_ ≤ 0.33 is denoted with “=”, 0.33 < BF_10_ < 3 with “?”, and BF_10_ ≥ 3 with “>” or “+”. L: left, M: middle, R: right, NF: non-fluent, F: fluent, C: corkscrew, H: human, P: pixelated, N: normal. The 4-way interaction was considered too complex to interpret and was not analyzed further.

**Table S6.** Means and Standard Deviations of the Base Rate Response

| **Left** | Non-Fluent | | Fluent | |
| --- | --- | --- | --- | --- |
|  | Pixelated | Normal | Pixelated | Normal |
| Corkscrew | 1.63 ± 0.86 | 1.71 ± 0.96 | 1.70 ± 0.89 | 1.63 ± 0.90 |
| Human | 1.99 ± 1.01 | 1.83 ± 0.93 | 1.82 ± 0.92 | 1.59 ± 0.82 |
| **Middle** | Non-Fluent | | Fluent | |
|  | Pixelated | Normal | Pixelated | Normal |
| Corkscrew | 2.64 ± 1.25 | 2.42 ± 1.20 | 2.66 ± 1.25 | 2.41 ± 1.25 |
| Human | 3.13 ± 1.47 | 2.52 ± 1.25 | 3.03 ± 1.45 | 2.39 ± 1.16 |
| **Right** | Non-Fluent | | Fluent | |
|  | Pixelated | Normal | Pixelated | Normal |
| Corkscrew | 2.25 ± 1.27 | 2.03 ± 1.24 | 2.23 ± 1.25 | 2.05 ± 1.26 |
| Human | 2.37 ± 1.25 | 2.16 ± 1.27 | 2.37 ± 1.41 | 1.93 ± 1.16 |
